# Changing subplate circuits: Early activity dependent circuit plasticity

**DOI:** 10.3389/fncel.2022.1067365

**Published:** 2023-01-11

**Authors:** Didhiti Mukherjee, Patrick O. Kanold

**Affiliations:** ^1^Department of Biomedical Engineering, Johns Hopkins University, Baltimore, MD, United States; ^2^Kavli Neuroscience Discovery Institute, Johns Hopkins University, Baltimore, MD, United States

**Keywords:** subplate neurons, development, spontaneous activity, sensory-driven activity, activity-dependent plasticity

## Abstract

Early neural activity in the developing sensory system comprises spontaneous bursts of patterned activity, which is fundamental for sculpting and refinement of immature cortical connections. The crude early connections that are initially refined by spontaneous activity, are further elaborated by sensory-driven activity from the periphery such that orderly and mature connections are established for the proper functioning of the cortices. Subplate neurons (SPNs) are one of the first-born mature neurons that are transiently present during early development, the period of heightened activity-dependent plasticity. SPNs are well integrated within the developing sensory cortices. Their structural and functional properties such as relative mature intrinsic membrane properties, heightened connectivity *via* chemical and electrical synapses, robust activation by neuromodulatory inputs—place them in an ideal position to serve as crucial elements in monitoring and regulating spontaneous endogenous network activity. Moreover, SPNs are the earliest substrates to receive early sensory-driven activity from the periphery and are involved in its modulation, amplification, and transmission before the maturation of the direct adult-like thalamocortical connectivity. Consequently, SPNs are vulnerable to sensory manipulations in the periphery. A broad range of early sensory deprivations alters SPN circuit organization and functions that might be associated with long term neurodevelopmental and psychiatric disorders. Here we provide a comprehensive overview of SPN function in activity-dependent development during early life and integrate recent findings on the impact of early sensory deprivation on SPNs that could eventually lead to neurodevelopmental disorders.

## Introduction

The efficiency of information processing in the sensory cortices relies on precise wiring and organization of underlying circuits that include billions of neurons and hundreds of thousands of synapses. Unfolding of genetic programs instruct a significant proportion of events in circuit development by establishing the first rough arrangement of connections which includes neural differentiation, migration, axon guidance, dendritic extension etc. (Polleux, 2005; Price et al., 2012; Diao et al., 2018; Kim and Kim, 2020). However, the maturation and assembly of rudimentary connections into functional networks require a dynamic interaction of intrinsic genetic programs with activity-dependent processes during prenatal and postnatal development (Cang and Feldheim, 2013; Choi, 2018; Simi and Studer, 2018).

**Figure 1:**
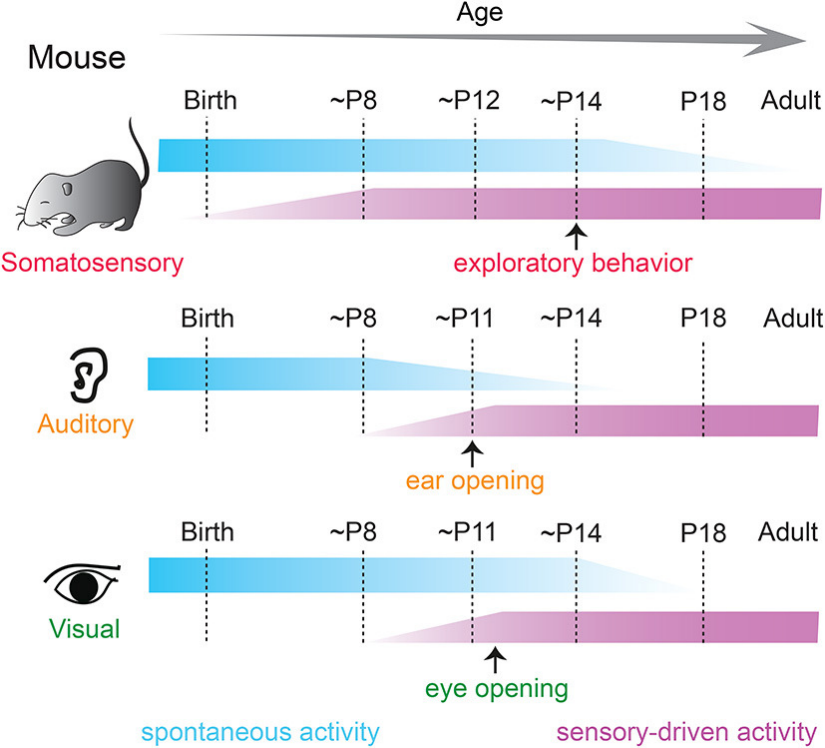
Spontaneous and sensory-driven activity in developing sensory systems in the mouse. Developmental trajectory of spontaneous (blue) and sensory-driven (magenta) activity in the somatosensory **(top)**, auditory **(middle)**, and visual **(bottom)** systems in the mouse. Sensory-evoked activity is observed before the onset of active sensory experience in all modalities. P, postnatal day.

A signature feature of neural activity during early development includes periodic “spontaneous” bursts of patterned activity within neural networks that are correlated among neighboring cells and are independent of external sensory stimulation (Galli and Maffei, 1988; Maffei and Galli-Resta, 1990; Katz and Shatz, 1996; Kirkby et al., 2013; Martini et al., 2021). Correlated spontaneous activity is observed in developing sensory cortices of all modalities in a variety of species (Feller, 1999; Khazipov and Luhmann, 2006; Blankenship and Feller, 2010; Luhmann et al., 2016) and is fundamental for sculpting and refinement of immature cortical connections (Thivierge, 2009; Ben-Ari and Spitzer, 2010; Kirkby et al., 2013; Levin, 2014) before the time when such changes can be driven by sensory experience from the environment (Figure 1).

Sensory-driven activity is the other major driver of cortical plasticity during development. Sensory experience from the environment substantially influences the structural and functional maturation of nascent neural structures once transduction mechanisms in the peripheral organs can convert environmental changes into electrical signals (Skaliora, 2002; Grubb and Thompson, 2004; Kolb and Gibb, 2011). The crude and usually overabundant early connections that are initially refined by spontaneous activity, are further elaborated by sensory experience such that orderly, stereotyped, and reliable connections are established for the proper functioning of the cortices. The effects of sensory experience on developing cortices are substantial during the classic “critical period”—a brief developmental time window of heightened plasticity, during which the developing brain is extremely sensitive to changes and can rapidly reorganize to adapt to the changing environment (Hubel and Wiesel, 1970; Barkat et al., 2011; Kreile et al., 2011; Dehorter et al., 2012; Erzurumlu and Gaspar, 2012). However, recent evidence suggests that cortical structures are malleable by environmental factors earlier than the onset of the critical period (Meng et al., 2021; Mukherjee et al., 2021; Tan et al., 2021).

During the orchestrated trajectory of sensory cortex development, there is an additional specialized population of neurons, known as the subplate neurons (SPNs). SPNs are one of the first born mature neurons in the sensory cortices that are transiently present during early corticogenesis (McConnell et al., 1989; Antonini and Shatz, 1990; Ghosh et al., 1990; Kostovic and Rakic, 1990; Kanold and Luhmann, 2010; Hoerder-Suabedissen and Molnar, 2015), specifically before the thalamic innervation of layer 4 neurons (Barkat et al., 2011; Erzurumlu and Gaspar, 2012) and are sharply reduced in number during postnatal development (Price et al., 1997; Torres-Reveron and Friedlander, 2007). Initially, SPNs were thought to serve as transient “waiting zones” for the developing thalamo-cortical projections (Allendoerfer and Shatz, 1994), however, subsequent clinical and experimental research has delineated an indispensable role of the SPNs in activity-dependent development of early cortical connections and function. For example, SPNs, despite their short life span, are essential elements in thalamo-cortical axon pathfinding, formation of the very first thalamo-cortical and cortico-cortical circuits, formation of cortical columnar structures, maturation of intracortical inhibitory connections, occurrence of ocular dominance columns and barrels (Ghosh et al., 1990; Ghosh and Shatz, 1992, 1993; Kanold et al., 2003; Kanold and Shatz, 2006; Friedlander and Torres-Reveron, 2009; Kanold and Luhmann, 2010; Kostovic and Judas, 2010; Tolner et al., 2012; Molnar et al., 2020). Peripheral perturbations can alter SPN connections and result in neurodevelopmental disorders (Nagode et al., 2017; Nicolini and Fahnestock, 2018; Sheikh et al., 2019, 2022; Luhmann et al., 2022).

While various aspects of SPNs morphology, origin, molecular diversity, function, and fate have been elaborately covered by previous review articles (Luhmann et al., 2009, 2018; Kanold and Luhmann, 2010; Molnar et al., 2020; Ohtaka-Maruyama, 2020), recent evidence revealed additional role of SPNs in activity-dependent plasticity of the developing sensory cortices during the earliest periods of postnatal development. The current review aims to (i) provide a comprehensive overview of SPN function in activity-dependent development of sensory cortices and (ii) integrate recent findings on the impact of early sensory deprivation on SPNs during early postnatal development that could eventually lead to neurodevelopmental and psychiatric disorders.

## Ultrastructural, morphological, and functional properties of subplate neurons: A brief overview

**Figure 2:**
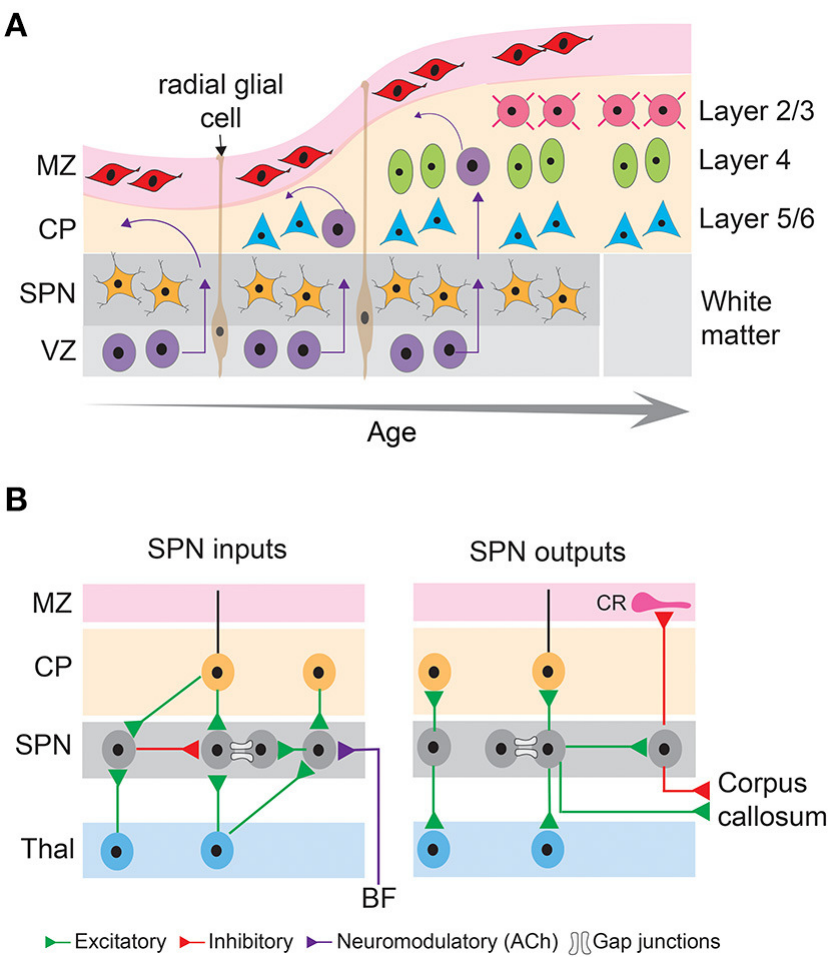
Basic principles of neocortical development and connectivity of subplate neurons. **(A)** Schematic diagram showing sequential generation of cortical layers. The earliest cohort of generated neurons comprises subplate neurons and cells in the marginal zone. Later generated neurons being born in the ventricular zone migrate along the processes of radial glial cells toward the pial surface and differentiate into different layers of the cortical plate, thus establishing the inside first-outside last orientation of the cortical layers. MZ, marginal zone; CP, cortical plate; SPN, subplate neurons; VZ, ventricular zone. **(B)** Schematic diagram showing inputs and outputs of subplate neurons that comprise excitatory (green), inhibitory (red) and neuromodulatory (purple) connections as well as gap junctional coupling. CR, Cajal-Retzius neuron; BF, basal forebrain; Thal, thalamus.

The earliest born SPNs, first discovered as a distinct layer in the human embryonic cerebral cortex (Kostovic and Molliver, 1974) reside at the bottom of the sensory cortices (Kostovic and Rakic, 1980) and are present in a variety of placental mammals including rodents, cats, ferrets, primates, and humans (Molnar et al., 2006). The majority of the SPNs are formed in the ventricular zone that initially populate the pre-plate and are subsequently separated into marginal zone and the basal SPNs by later born outwardly migrating neurons (Bystron et al., 2008). Cohorts of SPNs are also generated from the intermediate progenitors in the sub-ventricular zone (Vasistha et al., 2015) or migrate from the rostro medial telencephalic wall (Pedraza et al., 2014), suggesting SPNs contain heterogeneous subpopulations. Although SPNs are typically transient structures, and their numbers drastically decreases in the neonatal stage of development (Price et al., 1997; Torres-Reveron and Friedlander, 2007), evolutionary differences are observed in the ontogenetic fate of the SPNs. In primates, SPNs are thought to persist into adulthood as interstitial white matter neurons (Kostovic and Rakic, 1980, 1990), and in rodents they survive as layer 6B neurons (Aboitiz and Montiel, 2007; Marx et al., 2017) (Figure 2A). Species differences exist in the relative thickness of the SPN layers as well. In fact, there is an increase in thickness with evolution, with the highest thickness observed in humans and monkeys (Mrzljak et al., 1992; Molnar et al., 2006), suggesting that SPNs are not a vestige of early neuronal structures, instead they are a key element in higher intercortical connectivity (Aboitiz et al., 2005; Kanold and Luhmann, 2010).

A hallmark of SPNs is their relatively mature structural and functional properties. Due to their earlier generation and mature developmental stage, SPNs possess extensive axonal and dendritic extension and arborization (Friauf et al., 1990; Hanganu et al., 2001, 2002). Descending and ascending SPN dendrites extend up to the intermediate zone and cortical plate, respectively (Del Rio et al., 2000). SPN axons form short- and long-distance cortical and cortico-thalamic connections. The short distance but dense axon arborizations within the SPN layer form local circuits (Viswanathan et al., 2012; Meng et al., 2014). The long-distance axons serve as projection neurons to innervate the marginal zone/layer 1, cortical plate (axon collaterals to layer 4), thalamus, and distant cortical areas (McConnell et al., 1989, 1994; Friauf et al., 1990; De Carlos and O'Leary, 1992; Finney et al., 1998; Clancy and Cauller, 1999; Viswanathan et al., 2017). Whereas the projections to the cortical plate (including layer 4) arise mostly from glutamatergic SPNs (Friauf et al., 1990; Finney et al., 1998), GABAergic neurons are also present in the subplate zone and form long-distance cortico-cortical connections (Tomioka et al., 2005; Higo et al., 2007; Myakhar et al., 2011; Boon et al., 2019) (Figure 2B). These results suggest that SPNs could potentially link thalamocortical and corticothalamic circuits during development, however, there are limitations in their interpretation of the functionality of these connections. Most of these studies used anatomical, histological, immunohistochemical approaches and/or electrical stimulation, or glutamate uncaging methodologies to reveal anatomical or functional connectivity but did not measure the functional contribution of these connections to spontaneous or sensory-evoked activity. Selective activation, inactivation or silencing of the SPNs using optogenetic approaches combined with *in vivo* neural recording or imaging are needed to identify the developmental trajectory and function of SPN outputs. Such studies will depend on the development of new SPN-specific reporter mouse lines to analyze and characterize the connectivity of the different SPN subtypes and manipulate specific SPN subpopulations at key developmental stages. One challenge is that many subplate markers are only present at certain but not all developmental periods (e.g., mostly at later but not at early stages) and additionally that these markers can vary across cortical areas.

The presence of different subtypes of glutamate and GABA receptors in a variety of species suggest the presence of functional excitatory and inhibitory synapses to SPNs (Huntley et al., 1990; Meinecke and Rakic, 1992; Herrmann et al., 1994; Catalano et al., 1997; Viswanathan et al., 2012; Meng et al., 2014). In fact, it has been demonstrated that the majority of the afferent projections to the SPNs comprises glutamatergic inputs from the thalamus and neocortical areas and GABAergic input from within the SPN layer (Kostovic and Rakic, 1980; Luhmann et al., 2009). However, as detailed above SPNs are diverse, and it is unknown if all molecularly defined types of SPNs receive similar thalamic and intracortical inputs. In a recent study, sparse thalamic innervation was found in a genetically identified subpopulation of SPNs (*Lpar1-EGFP*) in the mouse somatosensory cortex during the first postnatal week after electrical and optogenetic activation of thalamic afferents in thalamocortical slices (Ghezzi et al., 2021). But it is unknown if all SPN subtypes receive thalamic input and if and how the input changes over the developmental period. More studies using selective SPN mouse lines are required to fully and functionally characterize the inputs to different types of SPNs during development. In addition to elaborated chemical synapses, SPNs are locally coupled *via* electrical synapses (Dupont et al., 2006) (Figure 2B).

Due to the morphological and neurochemical heterogeneity it has been challenging to identify markers specific to the SPNs. Recently an overlapping pattern of four well-defined SPN markers (Complexin3, CTGF, Nurr1, and Lpar1) and some additional markers (Moxd1, Tmem163 etc.) have been identified in subpopulations of SPNs in sauropsids and mammals (Hoerder-Suabedissen et al., 2009; Wang et al., 2011; Hoerder-Suabedissen and Molnar, 2013). These morphological, immunohistochemical and structural heterogeneity of the SPNs suggest their various evolutionary origins (Bruguier et al., 2020).

Electrophysiological recording from neocortical slices in different mammalian species revealed rather mature passive and active membrane properties of SPNs (Friauf et al., 1990; Luhmann et al., 2000; Hanganu et al., 2001, 2002; Aboitiz and Montiel, 2007; Hirsch and Luhmann, 2008; Moore et al., 2009). SPNs display an average membrane potential of −55 mV and membrane resistance higher than 1 GΩ, which allow even small postsynaptic currents in immature networks to trigger action potentials in SPNs (Luhmann et al., 2000; Hanganu et al., 2001; Zhao et al., 2009). Moreover, low resonance frequency and slow membrane time constant are suitable for the summation of recurring subthreshold synaptic inputs to the SPNs (e.g., thalamic bursts) (Sun et al., 2012). Compared to the immature cortical neurons in the marginal zone and/or cortical plate, SPNs display relatively mature electrophysiological properties, such as the largest amplitude in voltage-dependent sodium currents, repetitive and overshooting action potentials at frequencies exceeding 40 Hz in response to sustained depolarization by intracellular current injection (Luhmann et al., 2000, 2018; Hanganu et al., 2002; Dupont et al., 2006; Moore et al., 2009; Zhao et al., 2009; Kanold and Luhmann, 2010). Finally, SPNs are tightly coupled *via* electrical synapses/gap junction coupling and form a functional syncytium with cortical plate neurons that are implicated in activity-dependent columnar organization of cortical networks (Dupont et al., 2006; Luhmann et al., 2009, 2018). There is, however, a lack of knowledge on whether the gap junctional coupling between SPNs or between SPNs and other cortical neurons exhibit cell-type specificity. Given the heterogeneity in molecular markers and the efferent targets of SPNs, it will be compelling to address that to further characterize SPN functions in early development. Sophisticated techniques such as paired recordings, tracer coupling with analysis of network topography, imaging of ion sensitive dyes could be implemented to unravel cellular heterogeneity of SPN ion channels (Stephan et al., 2021). Together, the mature structural and electrical properties of the SPNs strongly suggest their active involvement rather than a passive transient waiting zone in cortical development.

## Spontaneous activity in early development: Involvement of subplate neurons

Spontaneous bursts of patterned activity—independent of apparent external input—are a hallmark of all developing sensory systems and are observed throughout the sensory pathways including the peripheral organs (e.g., retina, cochlea, and skeletal muscles), spinal cord, cerebellum, hippocampus, and neocortex in a variety of species (Blankenship and Feller, 2010; Feldt et al., 2011; Dehorter et al., 2012; Blumberg et al., 2013; Kirkby et al., 2013). The periphery, as well as the central sources might independently generate spontaneous activity (Siegel et al., 2012; Wang et al., 2015; Seabrook et al., 2017); however, their interactions are speculative. Periphery-generated spontaneous events are conveyed *via* the sensory thalamic nuclei to the developing cortices (Babola et al., 2018). Although the cellular mechanisms underlying the activity patterns change profoundly during specific stages of development (Luhmann et al., 2016), the endogenous bursts are generated within the network and are synchronized among neighboring cells.

The occurrence of spontaneous network activity coincides with the developmental period when the nascent neural circuits undergo vigorous sculpting and refinement of their connections and the initial formation of sensory maps (Katz and Shatz, 1996; Kirkby et al., 2013). In fact, the nature of the spontaneous activity patterns makes them suitable candidates to drive these developmental processes. For example, the overall prevalence of spontaneous activity and the repeated stimulation provided by the bursts are thought to provide the drive required for synaptic strengthening consistent with Hebbian principles of plasticity, the propagating nature and demarcated spatial boundaries could ensure that the sensory maps are retained across connected brain regions (Katz and Shatz, 1996; Eglen et al., 2003). Spontaneous endogenous activity is also implicated in programmed cell death during early development, which unfolds in a cell specific manner and precise temporal control to retain appropriate proportions of excitatory and inhibitory neurons and establish balanced neural circuits (Wong et al., 2018; Wong and Marin, 2019; Warm et al., 2022). Therefore, endogenous spontaneous activity is suitably placed at the forefront of activity-dependent sensory development.

**Figure 3:**
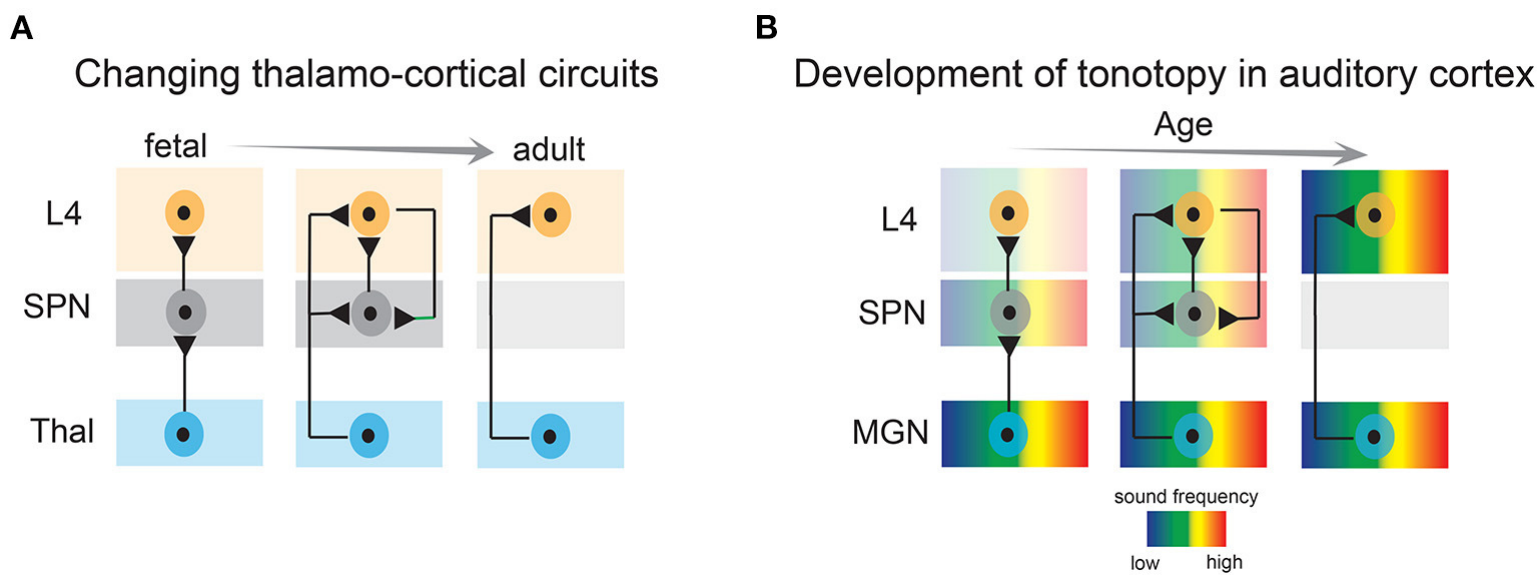
Developmental emergence of thalamo-cortical connections and emergence of tonotopy in the auditory cortex. **(A)** Subplate neurons convey thalamic activity to layer 4 neurons during development, whereas in adults, thalamic axons directly activate layer 4 neurons. L4, layer 4; SPN, subplate neurons; Thal, thalamus. **(B)** Tonotopic organization in the auditory cortex first emerges by projections to the subplate, and subsequently in layer 4. MGN, medial geniculate nucleus.

SPNs are well integrated within the developing sensory cortices and their structural and functional properties such as relative mature intrinsic membrane properties, heightened connectivity *via* chemical and electrical synapses, robust activation by neuromodulatory inputs—place them in an ideal position to serve as crucial elements in monitoring and regulating spontaneous endogenous network activity. For example, SPNs are the first targets of thalamocortical inputs before they innervate layer 4 (Friauf et al., 1990; Friauf and Shatz, 1991; Herrmann et al., 1994; Higashi et al., 2002; Molnar et al., 2003; Zhao et al., 2009; Barkat et al., 2011) (Figure 3A). Brief electrical stimulation of the thalamocortical axons reliably elicits fast excitatory postsynaptic currents in immature rat and cat SPNs (Friauf et al., 1990; Hanganu et al., 2002). SPNs in newborn rodents also receive intracortical presynaptic excitatory inputs from pyramidal neurons of the cortical plate and from within SPNs (Hanganu et al., 2002; Viswanathan et al., 2012). The later can sustain high frequency repetitive stimulation and is thought to augment thalamocortical inputs (Hirsch and Luhmann, 2008). SPNs in rodents also receive a GABAergic synaptic input from neighboring inhibitory neurons and from the cortical plate which likely has a depolarizing postsynaptic effect (Hanganu et al., 2002; Kilb et al., 2008; Ben-Ari and Spitzer, 2010). In addition to cortical and thalamocortical inputs, SPNs in primates and rodents receive a modest cholinergic innervation originating from the basal forebrain (Calarco and Robertson, 1995; Rakic, 1995; Mechawar and Descarries, 2001). Postsynaptic activation of acetylcholine receptors elicits heightened depolarization in SPNs mediated by α4/β2 receptors (Hanganu and Luhmann, 2004). Furthermore, as revealed by electrophysiological studies, SPNs are tightly coupled *via* electrical synapses and form a functional syncytium with the neighboring SPNs and the cortical plate neurons (Dupont et al., 2006). Therefore, with their extensive functional connectivity, SPNs are ideally suited for modulation and amplification of endogenous spontaneous activity originated peripherally as well as centrally.

In fact, a number of *in vitro* and *in vivo* experiments have identified spontaneous activity patterns in the SPNs during early development that are likely to modulate the activity-dependent maturation of cortical and thalamo-cortical structures (Luhmann et al., 2009; Kanold and Luhmann, 2010; Colonnese and Phillips, 2018). For example, SPNs in newborn rat somatosensory cortical slices exhibit substantial amount of postsynaptic current with different kinetics and pharmacological profiles suggesting different functional synaptic inputs onto SPNs (Hanganu et al., 2001). In response to depolarizing current injection, SPNs fire repetitive action potentials at frequencies up to 40 Hz in rodent cortex (Hanganu et al., 2001; Unichenko et al., 2015). Similar burst firing properties have been found in SPNs of postmortem human fetal brain tissues at gestational weeks 16–22 (Moore et al., 2009). Additionally, human fetal SPNs exhibit a tendency to generate spontaneous firing that includes plateau depolarizations and bursts of action potentials despite sparse synaptic inputs (Moore et al., 2011). This activity is thought to mechanistically rely on gap junctional coupling (Moore et al., 2014), as is also observed in the developing neocortex of postnatal mice (Singh et al., 2019).

GABAergic neurons in the subplate zone exhibit active involvement in regulating spontaneous oscillatory activity in the developing cortex. In neuronal cell cultures from embryonic rat cerebral cortex, a distinct population of large GABAergic neurons that reside in the subplate at the time of birth, were found as key elements in generating synchronous oscillatory neural firing. These neurons form interconnected network with extensive somato-dendritic innervation and axonal arborization and are thought to serve as integrating elements that synchronize neural activity by acquiring incoming intrinsic and extrinsic signals and distributing them effectively throughout the developing cortex (Voigt et al., 2001). The proposed roles of SPNs in synchronous network activity have been verified by *in vitro* neural recordings. Multichannel neural recording in acute neocortical slices of newborn mice demonstrated that electrical stimulation within the subplate zone in 800–1,000 μm slices elicits synchronized oscillatory activity (Sun and Luhmann, 2007). In fact, as observed in neocortical slices of fetal mice, SPNs might function as pacemakers or initiating zones of spontaneous activity that gradually takes the form of synchronized waves and propagate across both hemispheres (Lischalk et al., 2009). These observations were replicated in *in vivo* neural recording experiments. Extracellular recording of multiunit activity and local field potentials in newborn rat barrel cortex demonstrated that the current sinks of the majority of early spindle bursts and γ oscillations—distinct patterns of early network activity in all sensory cortices (Khazipov et al., 2004; Hanganu et al., 2006; Chipaux et al., 2013; An et al., 2014)—are located in the SPNs (Yang et al., 2009).

Mechanistically, cholinergic inputs to the SPNs play an important role in modulating spontaneous firing. Cholinergic activation of the SPNs triggers coordinated network activity in the neonatal somatosensory cortex. Upon cholinergic activation, non-synaptically released ambient GABA on the SPNs as well as gap junctional coupling facilitate the generation and propagation of cholinergic-dependent activity of the SPNs (Hanganu et al., 2007, 2009). Importantly, bath application of cholinergic agonists, such as carbachol induces propagating neural oscillations in spindle burst and γ frequency range in newborn rat thick cortical slices or in intact *in vitro* preparations of whole cortical hemisphere only when the SPNs are intact and are strongly synchronized within a cortical column *via* gap junctions (Dupont et al., 2006).

Similarly, *in vivo* intracortical EEG in neonatal rat somatosensory cortex demonstrated that selective removal of the SPNs in the S1 limb region abolishes endogenous and sensory-evoked spindle bursts. Additionally, selective removal of the SPNs in S1 barrel region prevents the characteristic barrel-like appearance (Tolner et al., 2012). Therefore, the active involvement of SPNs in the modulation of spontaneous activity suggests an integrative/instructive role of these neurons in early cortical development. Although these pioneering studies provide important data supporting functional involvement of SPNs in activity-dependent plasticity, there are limitations. These experiments involved subplate lesions using stereotaxic injection of kainic acid or immunotoxin saporin conjugated to the p75 neurotrophin receptors and resulted in a very focal SPN lesion (Kanold et al., 2003; Tolner et al., 2012). This approach is also limited to a small developmental time window, as the spatiotemporal subplate expression of the p75 receptors has a very short time window of ~2 days (e.g., P0–P1 in rats) (Koh and Loy, 1989). Finally, because these experiments were performed in genetically non-tractable species (cats and rats), it is not possible to delineate the involvement of specific SPN cell types in the observed phenomena. Therefore, it will be crucial to revisit these experiments using cell-type specific techniques to better understand the role of different subtypes of SPNs. Targeted deletion of specific SPN subtypes could be feasible using transgenic mouse lines if the driver line is expressing at young enough ages and if the driver line is exquisitely selective for subplate. For example, transgenic expression of diphtheria toxin receptors in such a SPN-specific Cre line, followed by intraperitoneal injection of the toxin could result in a selective lesion of the specific SPN subtype (Buch et al., 2005). However, it remains to be seen how efficient such a lesion would be e.g., what fraction of SPNs would be deleted. A potential downside of such a “modern” approach is that SPNs all over the cortex would be deleted (depending on their relative expression of Cre), in contrast to the high spatial selectivity of the older approaches (Kanold et al., 2003; Tolner et al., 2012).

Downstream effects of SPN oscillations have been speculated in cortical maturational processes, especially concerning the release of brain-derived neurotrophic factor (BDNF). BDNF is an indispensable mediator of activity-dependent plasticity (Wong et al., 2015) and is released from synaptically localized secretory granules following burst stimulation at 20–50 Hz (Lessmann et al., 2003). Because (a) SPNs can exhibit repetitive burst firing in the range of up to 40 Hz (Hanganu et al., 2009; Unichenko et al., 2015), (b) SPN ablation results in a paradoxical up-regulation in BDNF mRNA levels (Lein et al., 1999) and (c) consequently alters the formation and plasticity of ocular dominance columns (Lein et al., 1999; Kanold et al., 2003; Kanold and Shatz, 2006), it is possible that SPN-driven oscillations contribute to the local secretion of BDNF which strengthens synaptic connectivity in early neuronal ensembles. Because endogenous oscillations are actively involved in early programmed cell death (Wong et al., 2018; Wong and Marin, 2019; Warm et al., 2022), SPN oscillations could also mediate programmed cell death *via* activity-dependent BDNF release, as observed in organotypic slice cultures of neonatal cerebral cortex (Heck et al., 2008), and their dysfunction could have profound consequences in regulating early cortical plate cell survival and thus balance between excitatory and inhibitory neurons. Moreover, SPNs release neurotransmitters in paracrine, non-synaptic manner during oscillatory activity (Hanganu et al., 2009). This neurotransmitter release has been implicated in the migration of a large proportion of newly generated GABAergic interneurons from the medial ganglionic eminence and pyramidal neurons from the ventricular zone that must pass through the SPNs en route to the developing cortical plate (Kriegstein and Noctor, 2004). Together, these observations suggest that the spontaneous electrical events of SPNs play crucial roles in the activity-dependent development of cerebral cortices before coherent sensory stimuli from the periphery triggers activity (Luhmann et al., 2009; Kanold and Luhmann, 2010; Ohtaka-Maruyama, 2020). It is, however, not fully understood whether SPNs have an instructive role in early cortical oscillations in and of themselves, or they have a permissive effect on other network activity (e.g., GABAergic involvement), which themselves modulate oscillations. A recent study demonstrated that SPNs facilitate radial migration of interneurons in an activity-dependent manner, i.e., *via* transient synapse-like interaction (Ohtaka-Maruyama et al., 2018; Ohtaka-Maruyama, 2020). Because coherent activity of GABAergic interneurons is essential for proper maturation of cortical networks (Cossart, 2011, 2014; Kirmse and Zhang, 2022), it is possible that SPNs—by facilitating migration and maturation of interneurons and GABAergic signaling (Kanold and Shatz, 2006)—impose a permissive effect on the GABAergic interneurons, which themselves are involved in setting cortical oscillations. Further experiments by selectively preventing SPN mediated neural migration without affecting SPN activity will be able to reveal the mechanistic possibilities.

## Sensory-driven activity in early development: Involvement of subplate neurons

As development progresses, spontaneous activity becomes sparse and the dominant “burst” pattern switches to adult-like low amplitude desynchronized activity (Luhmann and Khazipov, 2018), which is complemented by progressively stronger input from the sensory periphery (Skaliora, 2002; Grubb and Thompson, 2004; Kolb and Gibb, 2011). Sensory input, in all modalities, reaches layer 4 of sensory cortices *via* thalamocortical projections (Hubel and Wiesel, 1962; Hunnicutt et al., 2014; Lopez-Bendito, 2018).

The impact of sensory experience on cortical development is the strongest during the sensitive and brief developmental epoch called the “classic critical period”. Although somewhat overlapping, the time window of critical period for each sensory modality is different. Whereas in the somatosensory system the critical period commences at birth, in the auditory and visual systems it coincides with the opening of ears and eyes, respectively, in altricial animals, which also marks the onset of active sensory experience (Hubel and Wiesel, 1970; Barkat et al., 2011; Kreile et al., 2011; Erzurumlu and Gaspar, 2012; Pedrosa et al., 2022). However, sensory cortices can be activated by external stimuli e.g., light, sound touch, etc. at incredible early ages, even before the thalamocortical projections innervate layer 4 neurons and the sensory organs are fully functional (Milh et al., 2007; Colonnese et al., 2010; Blumberg et al., 2013; Wess et al., 2017; Kaminska et al., 2018; Meng et al., 2021; Mukherjee et al., 2021; Tan et al., 2021), suggesting sensory inputs are relayed by some other transient structures that receive direct transmission from the thalamus. Importantly, during this early developmental time, the direct thalamocortical projections are constricted to the SPNs, which relay the ascending thalamic activity to layer 4 neurons (Zhao et al., 2009; Kanold and Luhmann, 2010; Barkat et al., 2011; Colonnese and Phillips, 2018) (Figure 3A). Although more *in vivo* electrophysiological evidence is required to support the assertion, based on existing results in several mammalian species it is reasonable to hypothesize that SPNs are the earliest substrates to receive early sensory-driven activity from the periphery and are involved in its transmission before the maturation of the direct adult-like thalamocortical connectivity.

About fifty years ago, neural recordings from fetal sheep brain revealed thalamocortical activation of deep cortical layers and surface positive responses after peripheral somatosensory stimulation (Molliver, 1967; Persson, 1973). Importantly, these responses were first noticeable at a fetal age of 55 days, a period in which fetal sheep have well defined SPNs (Astrom, 1967). At a comparable stage of development in fetal dogs (45 gestational days), when the cortical plate has no synapses, surface positive cortical responses are evoked upon peripheral somesthetic stimulation (Molliver and Van der Loos, 1970). These findings are supported by electrophysiological recording from SPNs in the visual cortex in fetal cat slices (Friauf et al., 1990). SPN responses after thalamic stimulation has also been observed in rodents. Long-lasting responses were recorded in thalamocortical slices by optical imaging in the SPNs of prenatal rats by embryonic day 18 after thalamic stimulation (Higashi et al., 2002). Similar results are observed in thalamocortical slices of mouse embryonic somatosensory cortex. As revealed by cortical calcium imaging, electrical stimulation of the ventral postero-medial nucleus of thalamus at embryonic day 17.5, evokes responses first in the SPNs, followed by responses in the cortical plate neurons (Anton-Bolanos et al., 2019). Based on these results it was suggested that deep synapses, likely at the SPNs are a substrate of early cortical evoked responses.

Recent studies have provided more direct evidence of the involvement of the SPNs in early sensory-driven activity. *In vivo* single unit recording from the ferret auditory cortex revealed that SPNs are responsive to peripheral sound stimulation before layer 4 neurons at postnatal day 23 when the ear canals are still closed and the thalamocortical projections directly innervate the SPNs. Moreover, electrode array recordings showed that early auditory responses exhibit a nascent topographic organization in the SPNs, suggesting topographic maps emerge in the SPNs before the onset of spiking responses in layer 4 neurons (Wess et al., 2017) (Figure 3B). It is important to note, that at these ages the ear canals were closed, and therefore sound stimuli were presented at 64–94 dB sound pressure level, which is louder than quiet natural sound stimuli (e.g., rustling leaves) but in the range of vocalizations of nest mates. Moreover, animals were anesthetized, which reduced cortical responsiveness and abolished spontaneous activity and only tonal stimuli were tested. *In vivo* experiments (imaging/neural recording) in awake animals (Meng et al., 2021; Mukherjee et al., 2021) with a wider range of naturalistic stimuli are still needed to fully characterize the sound-activation of SPNs at early ages. However, whatever the stimuli that drive SPNs are, as detailed below, raising animals in quiet environments altered SPNs circuits indicating that such stimuli are functionally effective. Similarly, activation of SPNs by naturalistic stimuli in other modalities remains unexplored so far. Moreover, to fully understand the functional coupling between thalamus, SPNs, and cortical neurons and to delineate the role of SPNs in modulation and transmission of peripheral input, simultaneous extracellular neural recording from thalamus, SPNs, and other cortical layers need to be performed. However, given the immaturity of cortex, high density recordings of large populations of isolated neurons will be challenging at young ages. Nonetheless, based on existing evidence, we can postulate that the developmental time window preceding the onset of the critical period (i.e., pre-critical period) is highly dynamic. Sensory experience from the periphery might have the potential to activate and sculpt SPN circuits during this period including topographic map formation (Figure 3B). Given these developmental dynamics, this period is now designated as a “proto-organizational period.”

A key question that immediately arises is “what sounds in the natural environment can activate SPNs during development?” Since ear canals are closed, externally generated sounds will have to be somewhat loud to overcome the attenuation in the ear canal. In contrast, self-generated sounds such as self-vocalizations will be less attenuated. Moreover, developing synapses exhibit high rates of adaptation to repeated/ongoing stimuli, and young neurons cannot sustain high firing rates. Therefore, it is unlikely that they respond to ongoing stimuli. Instead, rare, low frequency sounds are likely to show less adaptation and be most effective to activate SPNs (Hepper and Shahidullah, 1994). Some major intermittent sounds are produced by self- and other (e.g., mother, littermates)- generated vocalization. Because altricial animals are outside the womb and because their ear-canals are closed, other-generated vocalizations are likely to be attenuated. Therefore, it is intriguing to hypothesize that self-generated vocalizations could active SPNs and thus aid the development of the auditory system as do the self-generated muscle twitches during sleep in the somatosensory system (Blumberg et al., 2013, 2020). Such a scenario is not unreasonable to hypothesize as the classic work by Gilbert Gottlieb 50 years ago has demonstrated an important role of self-vocalization in auditory development (Gottlieb, 1971).

Similarly, in humans, external sounds are attenuated by the womb (Gerhardt et al., 1990) and the dominant sounds are those produced by the mother. Whereas sounds like breathing, heartbeat, digestive noises are ongoing with relatively constant spectral content, vocalization sounds, i.e., speech is irregular with varying frequency content and is likely to produce less adaptation. In fact, human fetuses and preterm infants can distinguish speech sound and non-speech sounds and respond to maternal voices before term suggesting sound experience shapes the fetal brain and complex auditory processing is possible in humans before term birth (Minai et al., 2017) pointing toward a possible involvement of the SPNs. However, one needs to consider that this is still indirect evidence as researchers recorded fetal heart rate in response to sound presentation and that there is no direct evidence of fetal brain activity to such stimuli. The prominence of the SPNs in mid-gestation, quantifiable number of synapses containing thalamic terminals in the SPNs, and absence of synapses in the cortical plate suggest that SPNs may be involved in cortical responses upon stimulation of the periphery and/or thalamus (Kostovic and Judas, 2010). Despite contradicting results from direct fMRI studies and limitations in analyzing the real generators in cortical responses in human fetuses, it seems reasonable to postulate that SPNs are engaged in physiological networks during the transition from fetal spontaneous activity in early preterm to sensory-driven activity in late preterm.

## Predicted roles of early subplate activity in cortical plasticity

Early spontaneous neural activity is indispensable for sculpting and refinement of immature cortical connections (Thivierge, 2009; Ben-Ari and Spitzer, 2010; Kirkby et al., 2013; Levin, 2014). The immature early connections that are initially refined by spontaneous activity, are further elaborated by sensory experience such that orderly, and functional connections are established for the proper functioning of sensory cortices (Skaliora, 2002; Grubb and Thompson, 2004; Kolb and Gibb, 2011; Molnar et al., 2020). A large number of experiments have demonstrated active involvement of SPNs in such plastic processes during early development (Kanold and Luhmann, 2010; Luhmann et al., 2018; Molnar et al., 2020).

Landmark studies by Carla Shatz and co-workers in the visual system of cats and ferrets have demonstrated that SPNs are critically involved in circuit formation and organization of cortical columns. For example, deletion of SPNs prevents axons originating from the lateral geniculate nucleus from identifying and innervating layer 4 neurons of the visual cortex, thereby impairing the formation of thalamocortical projections. Instead the axons continue to grow into the white matter (Ghosh et al., 1990). Similarly, prenatal ablation of SPNs prevents a subpopulation of cortical neurons from innervating the thalamus; thus, feedback corticothalamic projections are not properly formed (McConnell et al., 1989, 1994). SPNs play an important role in the formation of ocular dominance columns in the visual cortex. SPN deletion before the formation of ocular dominance columns prevents eye-specific segregation of LGN projections in the layer 4 of visual cortex (Ghosh and Shatz, 1992), resulting in weak visual responses that are also poorly tuned to orientation (Kanold et al., 2003); thereby suggesting that SPNs are required for anatomical refinement of thalamocortical projections that are essential for establishing the functional architecture of the visual cortex. Similarly, elimination of SPNs prevent spindle burst activity and the orchestration of the barrels in neonatal rat somatosensory cortex (Tolner et al., 2012), revealing a pioneer role of SPNs in the functional maturation of the somatosensory cortex.

Maturation and presence of inhibitory circuits are crucial in regulating critical period plasticity (Hensch, 2004). Focal lesion of SPNs prevents upregulation of genes involved in mature GABAergic transmission in layer 4 of visual cortex, therefore preventing the hyperpolarizing effect of GABA and affecting ocular dominance column plasticity (Kanold and Shatz, 2006). In a recent study using laser-scanning photo stimulation (LSPS) in cortical slices of postnatal mouse it was shown that glutamatergic signaling from SPNs are required for the maturation of GABAergic interneurons in the auditory cortex (Deng et al., 2017). Recent advances in transgenic technology have opened the opportunity to revisit these studies using cell-type specific approaches including DREADDs, optogenetics etc. However, such modern approaches also have limitations in that they depend on the early selective expression of Cre-recombinase, the uniform delivery and selective activation of agonists (e.g., dtx), or the selective induction of Cre in inducible strains by tamoxifen, DREADDs, all of which can also have side-effects that need to be controlled for (Martinez-Cerdeno et al., 2006; Goutaudier et al., 2020; Botterill et al., 2021). Nevertheless, despite the limitations of any technology, it will be important to start reinvestigating these questions using selective targeting of subtypes of SPNs in concert to established less selective techniques.

An important contribution of SPNs has also been demonstrated in neuronal migration in embryonic mouse cerebral cortex. Using a combination of techniques, it was shown that SPNs form transient synapses with migrating excitatory neurons just below the SPN layer and facilitate slow multipolar migration to a faster radial glial-guided migration (Ohtaka-Maruyama et al., 2018; Ohtaka-Maruyama, 2020). Given the role of GABAergic interneurons in setting cortical oscillations (Cossart, 2011, 2014), it is possible that SPNs provide a permissive effect on the migrating interneurons, which themselves are then involved in establishing oscillating activity.

Recently, secretory functions of SPNs have been implicated in cortical development. SPNs possess cellular morphology similar to cells known of secretory functions (Kondo et al., 2015). Moreover, some genes whose products are known to be secreted into the extracellular space, e.g., connective tissue growth factor (CTGF), Neuroserpin etc. are expressed in high levels in SPNs (Hoerder-Suabedissen et al., 2009; Hoerder-Suabedissen and Molnar, 2013; Kondo et al., 2015). In mice lacking CTGF in the forebrain, density of oligodendrocytes is increased, and the thickness of myelin sheath is reduced in the external capsule underneath layer 6b in young adult and middle aged mice, respectively, suggesting a secretory function of SPNs through the release of CTGF in cortical development (Yu et al., 2019).

As mentioned before, SPNs are highly active structures. For example, they elicit spatially confined spindle bursts and gamma oscillations, synchronize local columnar network via chemical and electrical synapses, respond to early peripheral stimuli even before thalamocortical projections innervate layer 4 of cortical plate (Dupont et al., 2006; Hanganu et al., 2009; Wess et al., 2017). Given the dynamic nature of the SPNs, it is plausible to predict that SPN-driven activity likely represents the functional prototype for the activity-dependent development of sensory cortices.

## Sensory perturbation in early development: Impact on subplate neurons

Responsiveness of the SPNs to sensory stimuli makes them vulnerable to sensory manipulations in the sensory periphery. Indeed, a broad range of early sensory deprivations in the periphery has been shown to alter SPN circuit organization and function in newborn animals. For example, presence of SPN neurites in the barrel fields of somatosensory cortex are altered after early postnatal whisker removal in newborn rats suggesting a sensory periphery-dependent integration of arrangement of SPN neurites during the period of barrel formation (Pinon et al., 2009).

**Figure 4:**
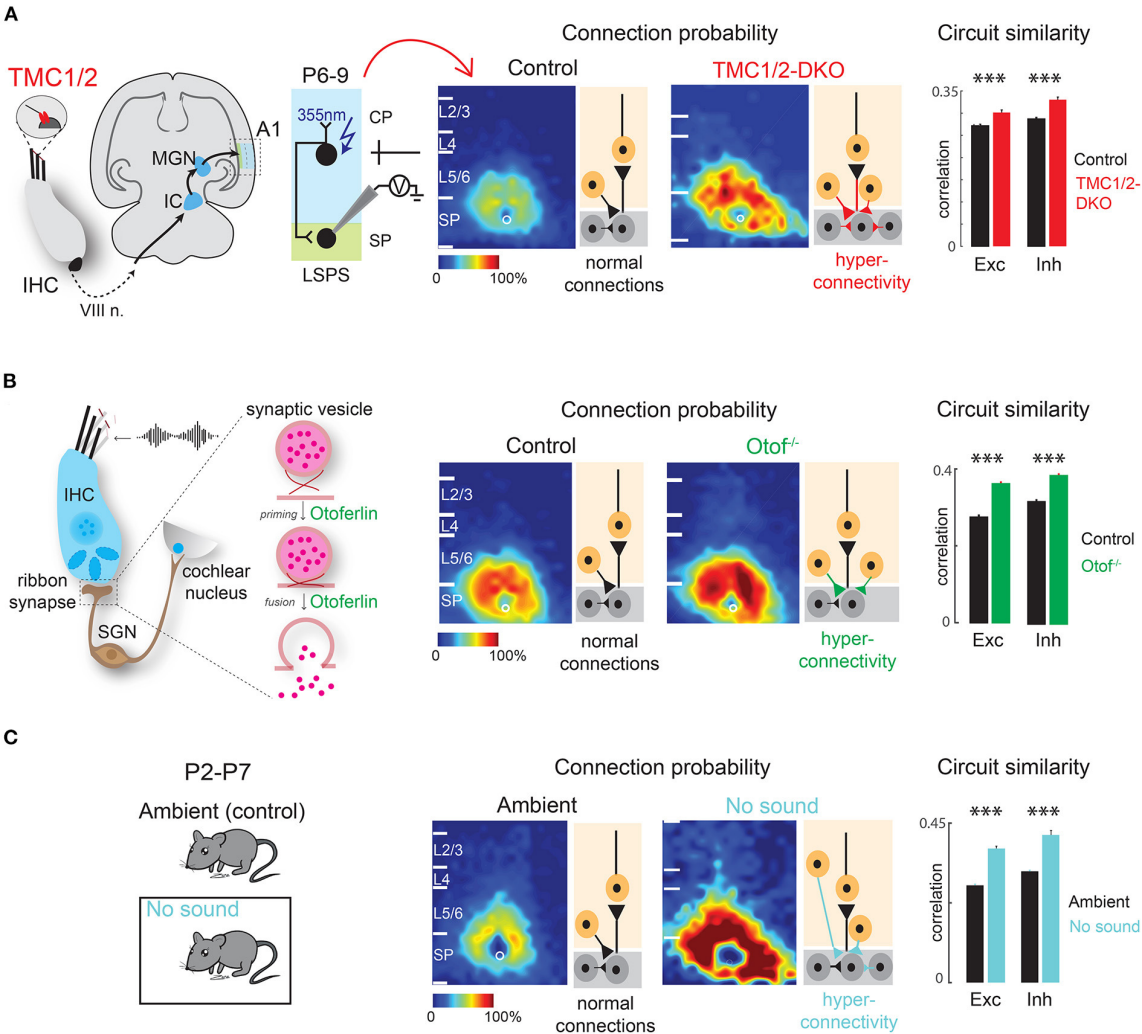
Early sensory deprivation alters subplate circuits in neonatal mice. **(A)** *Left:* Schematic diagram showing location of TMCs on tip link of cochlear inner hair cells. Hair cell activity is transmitted to the primary auditory cortex (A1) *via* auditory nerve (VIII n.), brainstem nuclei, inferior colliculus (IC), and medial geniculate nucleus (MGN). *Middle:* laser scanning photostimulation (LSPS) combined with whole cell patch clamp recording reveals hyperconnectivity of subplate neurons in TMC-1/2 DKO mouse pups. *Right:* Circuit similarity of both excitatory (Exc) and inhibitory (Inh) connections is higher in TMC-1/2 DKO mouse pups [from Meng et al. (2021)]. **(B)** *Left:* Schematic diagram showing the role of otoferlin in exocytosis at the ribbon synapses of the inner hair cells. *Middle:* Otof^−/−^ pups show hyperconnectivity of subplate neurons. Right: Otof^−/−^ pups show increased circuit similarity of both excitatory and inhibitory connections [from Mukherjee et al. (2021)]. **(C)** *Left:* Newborn pups were reared in a sound-attenuated chamber from P2–P7 (no sound). Control pups were reared in the normal ambient condition (ambient) in the colony. *Middle:* Pups show hyperconnectivity of subplate neurons in no sound condition. *Right:* Pups show increased circuit similarity of excitatory and inhibitory connections in no sound condition [from Meng et al. (2021)]. *p < 0.05.

Similarly, afferent intra-cortical circuits impinging on the SPNs are altered by peripheral manipulations. Significant changes in functional intra-cortical connectivity are observed in the SPNs of the auditory cortex in week-old mice that are born deaf/hearing impaired due to the absence of peripheral cochlear activity. Mice deficient in transmembrane channel-like proteins 1 and 2 [TMC1/2-DKO (double knockout)] never develop mechano-transduction in the cochlea and are born deaf (Kawashima et al., 2011; Pan et al., 2013; Askew et al., 2015). As revealed by LSPS combined with whole cell patch-clamp recording from SPNs in thalamocortical slices of week-old mice, the SPNs in TMC1/2-DKO pups receive increased amount of excitatory and inhibitory inputs from widespread areas in the cortical plate and from within the SPNs (Meng et al., 2021). Moreover, connections in TMC1/2-DKO pups are more stereotyped, meaning the spatial diversity of the circuits impinging on SPNs—a phenomenon that normally emerges with age (Meng et al., 2020)—is reduced in TMC1/2-DKO pups. These changes are reflected in globally more correlated spontaneous activity in TMC1/2-DKO pups as revealed by *in vivo* cortical imaging. Moreover, spontaneous release of excitatory and inhibitory neurotransmitters at the postsynaptic terminals on the SPNs are more frequent in the TMC1/2-DKO pups (Meng et al., 2021) (Figure 4A).

Similar results are obtained in mouse pups lacking the otoferlin-encoding gene OTOF (Otof^−/−^) in the ribbon synapses of inner hair cells in the cochlea (Roux et al., 2006; Grant et al., 2010; Pangrsic et al., 2010; Avraham, 2016). OTOF modulates neurotransmitter release at the ribbon synapses and thereby transmitting high fidelity auditory information to downstream structures (Heidrych et al., 2009; Ramakrishnan et al., 2009, 2014; Vogl et al., 2015; Michanski et al., 2019). As a result, Otof^−/−^ mice manifest impaired cochlear transmission and progressive hearing loss (Roux et al., 2006; Mukherjee et al., 2021). Consistent with TMC1/2-DKO pups, Otof^−/−^ pups show hyperconnectivity of excitatory and inhibitory inputs impinging on the SPNs of the auditory cortex from other cortical layers as well as higher circuit similarity. The hyperconnectivity is reflected in increased spatial correlation of spontaneous and sound-evoked activity of the cortex (Mukherjee et al., 2021) (Figure 4B). These results suggest that peripheral cochlear activity—which includes both spontaneous and sound-driven activity—is crucial for the circuit organization of the SPNs.

Additionally, there is direct evidence that lack of sound experience, in presence of cochlear spontaneous activity, alters SPN circuits in neonatal mice, even before the ear canals are open. Raising newborn mouse pups in a sound attenuated chamber in absence of any external ambient sound for 1 week results in hyperconnectivity of excitatory and inhibitory connections impinging on the SPNs with increased synaptic strength and less circuit diversity, mimicking the observations in TMC1/2-DKO and Otof^−/−^ pups (Meng et al., 2021) (Figure 4C). These observations collectively suggest that sound-driven activity from the periphery along with spontaneous cochlear activity are required for SPN circuit refinement. Therefore, not only the thalamo-cortical projections to the SPNs are functionally active during early development, but also the SPNs are the earliest substrates for experience-dependent plasticity, even before the onset of the classic critical period (Meng et al., 2021; Mukherjee et al., 2021). In general, lack of peripheral activity leads to intracortical hyperconnectivity of SPNs. The hyperconnectivity could be a compensatory mechanism to “fill in” for the under-developed thalamocortical projections to the SPNs. It will be intriguing to perform optogenetics combined with SPN slice recording to study thalamocortical afferents to SPNs after peripheral manipulations.

## Early environmental insult and pathophysiology of the subplate neurons

**Figure 5:**
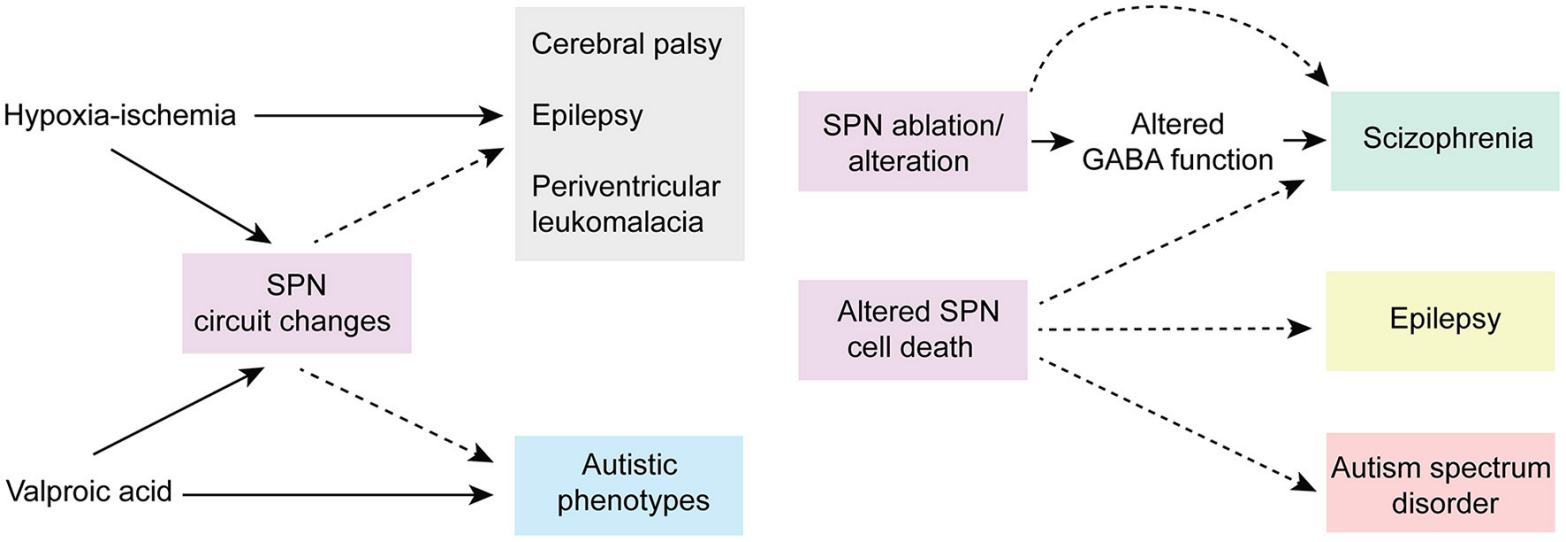
Predicted roles of SPN abnormality in neurodevelopmental disorders. Cartoon depicting possible involvement of SPN abnormality in neurodevelopmental and neuropsychiatric disorders based on existing experimental and clinical data. Solid arrows indicate direct experimental evidence. Dashed arrows indicate speculative assumptions based on experimental and indirect clinical evidence.

Given their location and sensitivity to environmental stimuli, SPNs are exposed to a wide range of environmental insults (i.e., drug, injury etc.). Importantly, animal research and clinical studies strongly indicate that early pathophysiological disturbances to SPNs are associated with several long term neurodevelopmental and psychiatric disorders (Kanold and Luhmann, 2010; Luhmann and Khazipov, 2018) (Figure 5). For example, hypoxic-ischemic brain injuries disrupt normal brain maturation and augments the risk of developing cerebral palsy, epilepsy, and periventricular leukomalacia (PVL) in human infants (du Plessis and Volpe, 2002; Ferriero, 2004; Volpe, 2012) suggesting underlying changes in cortical circuits. Experiments in neonatal rats have demonstrated circuit changes or total destruction of SPNs as a result of mild and severe hypoxia-ischemia, respectively (McQuillen et al., 2003; Failor et al., 2010; Sheikh et al., 2019), leading to abnormal cortical functional responses (Failor et al., 2010; Ranasinghe et al., 2015), white matter injury and deficits in motor behaviors as observed in human PVL (McQuillen et al., 2003). These results are also replicated in *in vitro* studies, where pronounced functional impairment of the SPNs is observed after oxygen-glucose deprivation (Albrecht et al., 2005). Importantly, in humans, the peak of SPN development coincides with the gestational age of the most prominent “window of vulnerability” to perinatal brain injury in preterm humans (McQuillen and Ferriero, 2005). In fact, immunohistochemical analysis on neonatal brains obtained postmortem from infants born at 25–32 weeks of gestation with white matter lesion has shown a significant loss of GABAergic neurons (Robinson et al., 2006), and activated microglial cells in the subplate zone (Pogledic et al., 2014). These observations together suggest that exposure to systemic and peripheral insults have the potential to alter SPN synaptic connectivity or histological damage to the SPNs, which may contribute to improper wiring in the developing brain leading to neurodevelopmental disorders. Indeed, hypoxia-ischemia injuries lead to altered SPN circuits and persistent changes in layer 4 circuits (Sheikh et al., 2019).

Altered connectivity is also a key feature in neuropsychiatric disorders including schizophrenia and autism spectrum disorder (Kostovic et al., 2011; Itahashi et al., 2014; Kambeitz et al., 2016) indicating SPN circuit changes could underlie the etiology of such disorders (Hadders-Algra, 2022) (Figure 5). In this line, it was shown using optical circuit mapping that the intracortical SPN connectivity is altered in the auditory cortex of week-old mice that are exposed prenatally to valproic acid (Nagode et al., 2017), a drug that has been shown to increase the incidence of autistic phenotypes in humans and in laboratory rodents (Williams et al., 2001; Roullet et al., 2010, 2013). In a mouse model of schizophrenia, the cytoarchitecture and number of subpopulations of neurons are altered in layer 6b, the remnant of SPNs in the adult rodent brain, implying a role of such aberrances in altered brain function in these mice (Tsai et al., 2020). Mechanistically, delayed maturation of GABA function is implicated in certain abnormalities in the prefrontal cortex (PFC) and manifestations of schizophrenia (Schmidt and Mirnics, 2015; Luhmann et al., 2018). Recently, it was shown in neonatal rats that SPN ablation results in decreased immunoreactivity of potassium-chloride transporter proteins (KCC2)—responsible for the maturation of GABAergic synapses—in the PFC, suggesting a possible involvement of early SPN lesion in development of schizophrenia (Lee and Rajakumar, 2022).

In addition to SPN injury or ablation, disturbances in the programmed cell death of the SPNs may also cause long term neurological deficits. An increased number of interstitial white matter neurons—presumed to be remnants of embryonic SPNs—have been reported in the frontal and temporal cortex and parahippocampal gyrus in patients with schizophrenia (Kirkpatrick et al., 1999; Eastwood and Harrison, 2005) and this has been attributed to alterations in programmed cell death (Akbarian et al., 1996). Existence of redundant local and long-range SPN synapses may disrupt cortical processing and may act as pacemaker regions for generation of epileptic brain activity (Bunney and Bunney, 2000; Luhmann et al., 2003). A large number of white matter neurons resembling SPNs have been identified in neocortical tissues of adult patients with temporal lobe epilepsy (Richter et al., 2016). SPNs outlived their normal lifespan in these cases, and in fact, surgical removal of these cortical malformations resulted in significant improvement in patients, suggesting SPNs are the epileptic focus (Luhmann et al., 2009, 2018; Richter et al., 2016). Frozen human postmortem tissue samples showed a large average density of NeuN (a neuronal marker)-positive neurons in layer 6 of patients with autism spectrum disorder, with cell morphologies consistent with SPNs, suggesting an abnormal initial population or a partial failure of apoptosis in SPNs aids atypical neural development in these patients (Avino and Hutsler, 2021). Moreover, there is evidence that hypoxia that occurs in most preterm infants leads to higher rate of programmed cell death (Hargitai et al., 2001). Because early network activity is known to regulate programmed cell death during postnatal development (Wong et al., 2018; Wong and Marin, 2019) and because SPNs are sensitive to hypoxia (Sheikh et al., 2019), one could speculate that preterm birth affects SPNs and their circuits, which in turn could impair their ability to facilitate endogenous activity, resulting in suboptimal survival signal to the cortex and increased cell death. Collectively, existing experimental, and clinical evidence strongly suggests a functional correlation between SPN pathophysiology and various neurodevelopmental and psychiatric disorders.

## Conclusions and future directions

Over the past few decades, we have gained a large amount of information from experimental outcome and clinical data on the functional involvement of SPN in the activity-dependent plasticity of the developing sensory cortices. Collectively, these evidence strongly suggest that SPNs are not merely a “transient waiting zone”, rather their transient presence with high dynamicity mark the “proto-organizational period” of the developing cortices. A large portion of the existing evidence, however, comes from experiments performed decades ago with the best methodologies available at those times and were mostly performed in animals not amenable to genetic manipulations (e.g., cats, ferrets, rats). As a result, those techniques lacked cell-type specificity. Thus, it is time we revisit those experiments using modern and more selective techniques. For further understanding the function of SPNs we propose the following future directions:

- Unambiguously mapping and characterizing SPN inputs and outputs to key targets using more selective approaches such as optogenetics.
- Validating results from the older SPN lesioning experiments by using selective ablation or targeted silencing methodologies. Newer techniques have the potential to reveal a more nuanced picture and uncover new findings that the older and less sensitive technologies failed to do.
- Determining if SPNs perform similar roles in different species.
- Directly identifying the functional coupling between thalamus, SPNs and cortical plate neurons across sensory systems to confirm the assertion that SPNs process early sensory stimuli in all sensory systems.
- Directly testing the assertions that SPNs amplify thalamic activity by using experimental systems studies or computational models. What would be the characteristics of such amplifier systems?
- Developing sensory cortices receive crossmodal multisensory input from other sensory pathways (Henschke et al., 2018), rendering the SPNs vulnerable to crossmodal alterations. Future experiments would seek to identify the effects of crossmodal manipulations on the structure, circuits, and function of the SPNs, and the understand the underlying neural mechanisms.
- Identifying the distinct roles of different subpopulations of SPNs in cortical plasticity.
- Identifying the effects of short-term and long-term peripheral manipulations on SPN structure, circuits, and function.
- Elaborately characterizing the functional contribution of SPNs in human development.

Although substantial progress has been made in this field of research over the past few decades, we need to acknowledge the inevitable challenges that the experiments impose as they need to be performed at delicate and early time points. The continuously developing new tools come from interests in studying mature systems and are routinely optimized to study adult organism, often disregarding development (e.g., cell density, size, physiological characteristics in spike-sorting algorithms, challenges in cranial window surgeries, transgenes which takes ~2 weeks to express, lack of Cre lines etc.). As much as we need to apply high-end techniques to go beyond the circumstantial/ indirect evidence to understand SPN functions, it is also high time we focus on optimizing tools and techniques to overcome the technical challenges to study early development.

## Data availability statement

The original contributions presented in the study are included in the article/supplementary material, further inquiries can be directed to the corresponding author.

## Author contributions

Both authors listed have made a substantial, direct, and intellectual contribution to the work and approved it for publication.
